# Are polymorphisms affecting serum urate, renal urate handling and alcohol intake associated with co-morbidities in gout cases? A case–control study using data from the UK Biobank

**DOI:** 10.1007/s00296-022-05148-7

**Published:** 2022-05-28

**Authors:** Gabriela Sandoval-Plata, Kevin Morgan, Abhishek Abhishek

**Affiliations:** 1 Academic Rheumatology, Clinical Sciences Building, City Hospital Nottingham, University of Nottingham, Hucknall Road, Nottingham, NG5 1PB UK; 2 Nottingham NIHR-BRC, Nottingham, UK; 3 Human Genetics, School of Life Sciences University of Nottingham, Nottingham, UK

**Keywords:** Gout, Uric acid, Hypertension, Comorbidity, Genetic variation

## Abstract

To examine the association between common comorbidities, eGFR and loci involved in the hyperuricaemia-gout transition. This study was conducted in people with gout from the UK Biobank. Logistic regression was used to examine the association between self-reported physician-diagnosed hypertension, diabetes, hypercholesterolemia and ischaemic heart disease (IHD) with the following variants: rs1260326(GCKR), rs16890979(SLC2A9), rs2231142(ABCG2), rs1229984(ADH1B) and rs2078267(SLC22A11) and adjusted for age, sex and 10-principal components. Linear regression was used to examine the association with eGFR. 7,049 participants with gout were included. After adjusting for multiple testing, there was a statistically significant positive association between urate lowering allele at SLC2A9 and hypertension, and negative association between urate raising allele at ABCG2 and hypertension (OR 1.17 and OR 0.86, respectively). Number of urate lowering risk alleles associated with hypertension [OR (95%CI) 1.13 (1.06–1.21)]. High eGFR associated with urate raising allele at rs2231142 (*β* = 1.38). The SNP in ADH1B that protects from alcohol excess showed a negative association with IHD (OR 0.53). Unlike in general population studies urate lowering genetic variants associate with hypertension in gout patients with dose–response. This may be due to high prevalence of other risk factors of hypertension such as obesity, poor diet etc. and needs validation in independent datasets.

**Supplementary Information:**

The online version contains supplementary material available at 10.1007/s00296-022-05148-7.

## Introduction

Gout is a common inflammatory arthritis and is associated with comorbidities such as hypertension, ischaemic heart disease (IHD) and chronic kidney disease (CKD) in observational studies [1, 2]. However, mendelian randomisation (MR) studies that have tested causality gave a conflicting picture with genetic urate variants associated with some comorbidities i.e. hypertension, IHD, hypercholesterolemia, but not with other comorbidities e.g. CKD and diabetes [3–7]. A meta-analysis of genome-wide association studies (GWAS) in 5 population-based cohorts of the Cohorts for Heart and Aging Research in Genome Epidemiology consortium (*n* = 28 283) did not find an association between genetic urate score and blood pressure, glucose, CKD, or coronary heart disease [8]. Other studies have reported a negative association between urate genetic risk score and blood pressure [9]. However, these studies were conducted in the general population.

Recent GWAS identified genetic variants in urate transporters (ABCG2, SLC2A9, SLC22A11) and metabolic genes (GCKR, ADH1B) to be associated with the transition from hyperuricaemia to gout [10], and several of these associated with serum urate (SU) and gout in previous studies [11–15]. Whether these genetic variants are associated with comorbidities in people with gout has not been examined before. This is an important question as comorbidities are common in gout and genetic variants involved in hyperuricemia-gout transition have associations beyond urate handling. For instance, the A allele at rs1229984 in *ADH1B* is associated negatively with IHD, while the T allele at rs1260326 in *GCKR* is associated with high triglyceride and low blood sugar levels in the general population [16, 17].

Thus, the purpose of this study was to examine the association between hypertension, diabetes, hypercholesterolemia, IHD, and estimated glomerular filtration rate (eGFR) and lead single nucleotide polymorphisms (SNPs) in urate transporter (rs16890979, rs2231142, and rs2078267 in SLC2A9, ABCG2, SLC22A11 respectively) and other genes (rs1260326 and rs1229984 in GCKR and ADH1B, respectively) associated with hyperuricaemia-gout transition.

## Methods

### Data source

This study was conducted using data from the United Kingdom (UK) Biobank (UK Biobank Project ID 45,987, approval date: 18th April 2019). The UK Biobank is a large population-based prospective study. Participants aged 40–69 years were recruited between 2006 and 2010 across England, Wales and Scotland. Data were collected on sociodemographic characteristics, lifestyle information, health status, family medical history and cognitive function. Physical and functional measurements were recorded and biological samples were collected for biomarkers measurements and genetic analyses. UK Biobank has approval from the North West Multi-Centre Research Ethics Committee (REC reference 16/NW/0274). This study did not involve re-contacting participants and no separate ethics approval was required. Details about recruitment and sample processing are described elsewhere [18].

### Subjects

This research included UK Biobank participants with gout with genome-wide genotype data available (*n* = 7049). Exclusion criteria were non-European ethnicity, self-reported and genetic sex mismatches, kinship coefficients equivalent to second degree (or greater) relatives, call-rate < 90% and heterozygosity outliers [defined as ± 3 standard deviations (SD) from the mean]. Gout was defined as present if participants met any of the following criteria: self-reported physician diagnosed gout, urate-lowering treatment prescription without a hospital diagnosis of lymphoma or leukaemia, or a primary or secondary hospital diagnosis of gout using the International Classification of Diseases (ICD)-10 codes M10 (Gout), M100 (Idiopathic gout), M101 (Lead induced gout), M102 (Drug Induced Gout), M103 (Gout Due to Renal Impairment), M104 (Other Secondary Gout) and M109 (Gout, unspecified).

#### Study-design

##### Cases-control study

Separate case–control analyses were performed to examine the association between hypertension, diabetes mellitus, hypercholesterolemia and IHD with candidate SNPs. Cases were participants with hypertension, diabetes mellitus, hypercholesterolemia and IHD, respectively while controls did not have the index comorbidity. Comorbidities were categorised as present if they were self-reported as physician diagnosed.

##### Cross-sectional study

A cross-sectional study was conducted to examine the association between eGFR calculated using the CKD-EPI equation and candidate SNPs.

### SNP selection and genotyping

A recent GWAS of gout cases and hyperuricaemic controls reported loci associated with hyperuricemia-gout transition [10]. From these loci, we selected the following key SNPs to perform the association analyses: rs1260326 (GCKR), rs16890979 (SLC2A9), rs2231142 (ABCG2), rs1229984 (ADH1B) and rs2078267 (SLC22A11). These SNPs were obtained from the directly genotyped UK Biobank data. Details about genotyping and the internal quality control (QC) procedures performed by the UK Biobank have been described previously [18]. The variants were evaluated to verify they did not deviate from Hardy–Weinberg equilibrium and had a call-rate > 95%.

### Statistical analyses

Number (%) and SD were calculated for descriptive purposes. Independent sample *t* test and *Χ*^2^ test were used for comparing continuous and categorical data respectively. Logistic regression was used to examine the allelic association of each SNP included in the study with comorbidities of interest. The number of risk alleles in rs2231142 and rs16890979 were added (range 0–2) and included as a continuous independent variable in logistic regression to examine their association with hypertension. Linear regression was used to determine the allelic association between SNPs and eGFR. Analyses were adjusted for age, sex, body mass index (BMI) and the first 10 principal components (PCs). Adjusted odds ratios (aORs) and 95% confidence intervals (CI), or Beta coefficients and standard error (SE) were calculated as appropriate. Bonferroni correction was used to account for multiple testing. The obtained *p* values were multiplied by 25 to obtain Bonferroni corrected p values and, *p*_corr_ < 0.05 was regarded as statistically significant. Data were managed and analysed using Stata/MP, and PLINK V.1.9 was used for QC analyses.

## Results

Data for 7049 gout cases (6479, 91.9% men) were included. Their mean (SD) age and body mass index was 60.09 (6.86) years and 30.74 (4.95) kg/m^2^ respectively. 2411 (34.2%), 1940 (27.5%), 1589 (22.5%), 406 (5.8%) and 693 (9.8%), participants self-reported alcohol consumption daily, 3–4 times/week, 1–2 times/week, < 1 per week and never or on special occasions respectively. The prevalence of current, ex- and non-smokers was 8.8% (*n* = 617), 50.3% (*n* = 3546), and 40.6% (*n* = 3560), respectively. The demographic characteristics of cases with index comorbidity and controls without comorbidity were comparable except for younger age in cases with diabetes compared to controls, and higher SU in cases with IHD (Supplementary Tables S1–S4).

There was an association between the T allele at rs16890979 (SLC2A9 gene) and hypertension with aOR (95%CI) 1.17 (1.06–1.29), *p*_corr_ 0.0355 (Table 1). There was also a negative association between T allele at rs2231142 (ABCG2 gene) and hypertension with aOR (95%CI) 0.86 (0.79–0.93), *p*_corr_ 0.0080 (Table 1). In previous studies, the T allele at rs16890979 and the G allele at rs2231142 have been associated with low SU level [8]. Thus, we defined the T allele at rs16890979 polymorphism and G allele at rs2231142 polymorphism [the G allele at rs2231142 associated with hypertension with aOR (95%CI) 1.16 (1.07–1.1.27)] as the risk alleles for exploring their additive effects on the association with hypertension. We observed that each additional risk allele increased the aOR (95%) for hypertension by 1.13 (1.06–1.21), with no risk allele referent (Table 2). The ORs increased progressively but were not significantly different for 1 or 2 alleles (Table 2). There was a statistically significant negative association between T allele at rs1229984 (ADH1B gene) and IHD [aOR (95%CI) 0.53 (0.38–0.74)], *p*_corr_ 0.0047. eGFR associated with T allele at rs2231142 (ABCG2 gene) with beta coefficient (SE) 1.38 (0.374), *p*_corr_ 0.0055. Additionally, there was a nominal association between T allele at rs1229984 and diabetes and eGFR associated with T allele at rs12603262 (GCKR gene) that was non-significant on accounting for multiple tests (Table 1).

**Table 1:** Association between comorbidities and lead SNPs

Single nucleotide polymorphism^a^	Chromosome number	Base pair	Gene	A1	Adjusted odds ratio (95% confidence interval)^b^	*p* value^c^
Hypertension						
rs1260326	2	27,730,940	GCKR	T	0.94 (0.87–1.00)	1.00
rs16890979	4	9,922,167	slc2a9	T	1.17 (1.06–1.29)	0.0355
rs2231142	4	89,052,323	ABCG2	T	0.86 (0.79–0.93)	0.0080
rs1229984	4	100,239,319	ADH1B	T	0.83 (0.69–0.99)	0.950
rs2078267	11	64,334,114	SLC22A11	C	0.97 (0.90–1.03)	1.00
Diabetes						
rs1260326	2	27,730,940	GCKR	T	0.91 (0.82–1.01)	1.00
rs16890979	4	9,922,167	SLC2A9	T	1.16 (1.01–1.34)	0.90
rs2231142	4	89,052,323	ABCG2	T	0.92 (0.81–1.05)	1.00
rs1229984	4	100,239,319	ADH1B	T	0.61 (0.43–0.85)	0.10
rs2078267	11	64,334,114	SLC22A11	C	1.03 (0.93–1.14)	1.00
Hypercholesterolemia						
rs1260326	2	27,730,940	GCKR	T	1.05 (0.97–1.14)	1.00
rs16890979	4	9,922,167	SLC2A9	T	1.06 (0.96–1.18)	1.00
rs2231142	4	89,052,323	ABCG2	T	0.99 (0.90–1.09)	1.00
rs1229984	4	100,239,319	ADH1B	T	0.87 (0.70–1.07)	1.00
rs2078267	11	64,334,114	SLC22A11	C	0.95 (0.88–1.02)	1.00
Ischaemic heart disease						
rs1260326	2	27,730,940	GCKR	T	1.04 (0.94–1.15)	1.00
rs16890979	4	9,922,167	SLC2A9	T	1.14 (0.99–1.30)	1.00
rs2231142	4	89,052,323	ABCG2	T	0.92 (0.81–1.04)	1.00
rs1229984	4	100,239,319	ADH1B	T	0.53 (0.38–0.74)	0.0047
rs2078267	11	64,334,114	SLC22A11	C	0.86 (0.78–0.95)	0.10
eGFR^e^				aβ (SE)^b,d^		
rs1260326	2	27,730,940	GCKR	T	0.902 (0.305)	0.10
rs16890979	4	9,922,167	SLC2A9	T	− 0.816 (0.426)	1.00
rs2231142	4	89,052,323	ABCG2	T	1.38 (0.374)	0.0055
rs1229984	4	100,239,319	ADH1B	T	0.397 (0.816)	1.00
rs2078267	11	64,334,114	SLC22A11	C	0.449 (0.299)	1.00

^a^The minor allele frequencies (MAF) for the SNPs are: rs1260326 MAF = 0.435; rs16890979 MAF = 0.145; rs2231142 MAF = 0.202; rs1229984 MAF = 0.035; and rs2078267 MAF = 497

^b^Adjusted for age, sex, body mass index and 10 principal components

^c^Bonferroni corrected

^d^Adjusted beta-coefficient (standard error)

^e^Estimated glomerular filtration rate

**Table 2:** Prevalence of hypertension according to number of risk alleles*

rs16890979/rs2231142 alleles	Hypertension	Odds ratio (95% confidence interval)	Adjusted odds ratio (95% confidence interval)^a^
	Prevalence %, *n*		
CC/TT	54.5, 102/187	1	1
CC/TG or TC/TT	53.9, 960/1780	0.98 (0.72–1.32)	0.86 (0.63–1.18)
CC/GG or TC/TG or TT/TT	58.0, 2202/3796	1.15 (0.86–1.55)	0.98 (0.72–1.34)
TC/TG or TT/TG	62.4, 738/1182	1.39 (1.01–1.89)	1.18 (0.85–1.63)
TT/GG	65.9, 56/85	1.61 (0.94–2.74)	1.40 (0.81–2.44)

^a^Adjusted for age, sex, body mass index and 10 principal component

*Genotype data were missing for 19 participants

## Discussion

This study examined the genetic factors associated with comorbidities in gout. It reported a statistically significant negative association between SNPs in the ABCG2 and SLC2A9 genes that are associated with high SU and hypertension with evidence of dose response. There was a negative association between SNP in ADH1B gene and IHD, and an association between T allele at ABCG2 polymorphism associated with high urinary urate excretion, and higher eGFR [20].

The association between genetic variants associated with low SU and hypertension is counterintuitive and not consistent with most of the results of epidemiological and MR studies that reported an association between gout and hypertension, and urate variants and hypertension and systolic blood pressure, respectively [2–4, 7]. However, a study by Sumpter et al. reported a negative association of a genetic risk score mostly driven by variants in ABCG2 and ADH1B [OR 0.88 (0.78–0.99); OR 0.69 (0.54–0.89) respectively] with the presence of any comorbidity in gout [21]. Hyperuricaemia induced by urate under-excretion was observed to be causal in animal models of hypertension and further research is required to understand why this is different in people with gout [22]. However, in healthy adults, acute infusion of uric acid did not increase blood pressure or cause any cardiovascular haemodynamic effects [23]. Nevertheless, the results of the current study imply that genotyping for polymorphism at the SLC2A9 and ABCG2 genes may be useful when counselling gout patients about risk of hypertension and the need to take preventative measures.

Our finding of a negative association between polymorphisms in ADHB1 gene and IHD is consistent with previous reports in the general population and may be due to a protective effect on excessive alcohol intake and/or binge drinking from this polymorphism [24]. It underlines the importance of the need to advice to reduce excess alcohol intake in people with gout.

The association between T allele at rs2231142 and gout differs across ethnicities [25]. Moreover, the T allele at rs2231142 is more common in the South-East Asian ethnicities than in the white Caucasian population [26–28]. Converse is true for the G allele at rs2231142. Further research in other ethnicities is required to confirm if these findings of an association between SU lowering alleles and hypertension holds true across populations.

Strengths of this study included large sample size, evaluation of dose response, use of eGFR values as continuous variables rather than categorical CKD stages, recruitment from the general population, and correction for multiple testing. However, the study had several limitations. Comorbidities were self-reported as being physician diagnosed. This has the potential to affect the strength of association due to biassed recall and is a key limitation. However, participants in UK Biobank reported their health conditions with the support of a research nurse present. Moreover, data on these covariates have been used extensively in research using UK Biobank data. In a validation study, self-reported physician diagnosed comorbidities in the UK Biobank had the same association with mortality as comorbidities ascertained using hospitalisation records alone [29]. Similarly, as information on the presence of monosodium urate crystals, tophi, bone erosions and other radiographic findings typical of MSU crystal deposits are not included in the UK Biobank, gout status could only be ascertained using self-report of physician diagnosis, ULT prescription and hospitalisation codes. Although not 100% specific, this case definition strategy has high sensitivity and precision for detecting association in genetic epidemiological studies of gout [30]. Inclusion of hospital discharge diagnosis increased the potential for this definition to include cases with more severe gout that may not have been diagnosed in primary-care or treated with ULT. Participants were not required to meet the 2015 American College of Rheumatology (ACR)/European League Against Rheumatism (EULAR) gout classification criteria as the UK Biobank baseline data collection occurred between 2006 and 2010, before the ACR/EULAR gout classification criteria had been published. This has the potential to reduce the validity of the results and our findings ought to be confirmed in independent datasets of gout cases meeting the ACR/EULAR classification criteria.

In conclusion, this study reported genetic factors associated with comorbidities in people with gout. The results may be used to risk-stratify gout cases for closer monitoring of comorbidities and for the use of primary prevention strategies. These results should also be validated in independent datasets, preferably in people from other ethnicities.

## Supplementary Information

Below is the link to the electronic supplementary material.

Supplementary file1 (DOCX 31 KB)

## Data Availability

Data used in this study were made available to the study team by the UK Biobank under study specific licence that does not allow us to make the data publicly available. Raw data used in the study may be obtained form the UK Biobank on application.

## Declarations

### Patient and public involvement

Patients or public were not involved in the design, report, or dissemination of our research.
