# Supplementary material for: Are polymorphisms affecting serum urate, renal urate handling and alcohol intake associated with co-morbidities in gout cases? A case–control study using data from the UK Biobank
Source: Rheumatol Int. 2022 May 28;42(9):1617–22. doi: 10.1007/s00296-022-05148-7 (PMC9349305; doi:10.1007/s00296-022-05148-7)
Supplement: Supplementary file 1 — Supplementary file1 (DOCX 31 KB) [file 296_2022_5148_MOESM1_ESM.docx]

Supplementary Table S1. Demographic and lifestyle characteristics for UK Biobank participants with gout, with and without hypertension.

|  | Hypertension | Hypertension |
| --- | --- | --- |
|  | n=4065 | n=2984 |
|  | + | - |
| Age (mean (SD)), years | 58.82 (8.82) | 59.03 (7.51) |
| Male sex (n (%)) | 3,730 (91.76) | 2,749 (92.12) |
| BMI (mean (SD)), kg/m^2^ | 29.53 (4.47) | 29.76 (4.78) |
| SU (mean (SD)), mg/dL | 6.64 (1.58) | 6.86 (1.84) |
| Alcohol intake (n (%))* |  |  |
| Never or only on special | 398 (9.79) | 295 (9.89) |
| <1/week | 237 (5.83) | 169 (5.66) |
| 1-2/week | 936 (23.03) | 653 (21.88) |
| 3-4/week | 1,105 (27.18) | 835 (27.98) |
| Daily or almost daily | 1,381 (33.99) | 1,030 (34.52) |
| Smoking (n (%))* |  |  |
| Non-smoker | 1,675 (41.21) | 1,185 (39.71) |
| Ex-smoker | 2,005 (49.32) | 1,541 (51.64) |
| Current-smoker | 371 (9.13) | 246 (8.24) |

*The following data were missing: alcohol intake for 10 (0.14%), and smoking status for 26 (0.37%).

Supplementary Table S2. Demographic and lifestyle characteristics for UK Biobank participants with gout, with and without diabetes.

|  | Diabetes | Diabetes |
| --- | --- | --- |
|  | n=834 | n=6215 |
|  | + | - |
| Age (mean (SD)), years | 56.68 (9.21) | 59.20 (8.03) |
| Male sex (n (%)) | 762 (91.37) | 5717 (91.98) |
| BMI (mean (SD)), kg/m^2^ | 29.78 (4.63) | 29.63 (4.63) |
| SU (mean (SD)), mg/dL | 6.74 (1.66) | 6.75 (1.72) |
| Alcohol intake (n (%))* |  |  |
| Never or only on special | 97 (11.63) | 596 (9.59) |
| <1/week | 48 (5.76) | 358 (5.76) |
| 1-2/week | 206 (24.70) | 1383 (22.25) |
| 3-4/week | 194 (23.26) | 1746 (28.09) |
| Daily or almost daily | 289 (34.65) | 2122 (34.14) |
| Smoking (n (%))* |  |  |
| Non-smoker | 80 (9.59) | 537 (8.64) |
| Ex-smoker | 409 (49.04) | 3137 (50.47) |
| Current-smoker | 343 (41.13) | 2517 (40.50) |

*The following data were missing: alcohol intake for 10 (0.14%), and smoking status for 26 (0.37%).

Supplementary Table S3. Demographic and lifestyle characteristics for UK Biobank participants with gout, with and without hypercholesterolemia.

|  | Hypercholesterolemia | Hypercholesterolemia |
| --- | --- | --- |
|  | n=1953 | n=5096 |
|  | + | - |
| Age (mean (SD)), years | 58.07 (8.25) | 59.24 (8.16) |
| Male sex (n (%)) | 1785 (91.40) | 4694 (92.11) |
| BMI (mean (SD)), kg/m^2^ | 30.99 (5.04) | 29.13 (4.36) |
| SU (mean (SD)), mg/dL | 6.86 (1.74) | 6.70 (1.70) |
| Alcohol intake (n (%))* |  |  |
| Never or only on special | 214 (10.95) | 479 (9.40) |
| <1/week | 108 (5.53) | 298 (5.85) |
| 1-2/week | 436 (22.32) | 1153 (22.63) |
| 3-4/week | 516 (26.42) | 1424 (27.94) |
| Daily or almost daily | 675 (34.56) | 1736 (34.06) |
| Smoking (n (%))* |  |  |
| Non-smoker | 815 (41.73) | 2045 (40.13) |
| Ex-smoker | 950 (48.64) | 2596 (50.94) |
| Current-smoker | 184 (9.42) | 433 (8.50) |

*The following data were missing: alcohol intake for 10 (0.14%), and smoking status for 26 (0.37%).

Supplementary Table S4. Demographic and lifestyle characteristics for UK Biobank participants with gout, with and without Ischaemic heart disease.

|  | IHD | IHD |
| --- | --- | --- |
|  | 956 | 6093 |
|  | + | - |
| Age (mean (SD)), years | 58.09 (8.84) | 59.03 (8.12) |
| Male sex (n (%)) | 870 (91.01) | 5609 (92.06) |
| BMI (mean (SD)), kg/m^2^ | 29.95 (4.48) | 29.61 (4.64) |
| SU (mean (SD)), mg/dL | 7.42 (1.48) | 6.66 (1.72) |
| Alcohol intake (n (%))* |  |  |
| Never or only on special | 99 (10.36) | 594 (9.75) |
| <1/week | 55 (5.75) | 351 (5.76) |
| 1-2/week | 212 (22.18) | 1377 (22.60) |
| 3-4/week | 242 (25.31) | 1698 (27.87) |
| Daily or almost daily | 347 (36.30) | 2064 (33.87) |
| Smoking (n (%))* |  |  |
| Non-smoker | 396 (41.42) | 2464 (40.41) |
| Ex-smoker | 471 (49.27) | 3075 (50.47) |
| Current-smoker | 88 (9.21) | 529 (8.68) |

*The following data were missing: alcohol intake for 10 (0.14%), and smoking status for 26 (0.37%).
